# Opportunities and pathways for patient and public involvement

**DOI:** 10.1111/hex.12878

**Published:** 2019-03-25

**Authors:** Parisa Aslani

**Affiliations:** ^1^ Medicines Use Optimisation, The University of Sydney School of Pharmacy, Faculty of Medicine and Health The University of Sydney Camperdown New South Wales Australia

To provide effective health‐care services to patients and the public, it is imperative that health‐care professionals, policy makers and other key stakeholders involve and engage patients and the public at every step of the way. To conceptualize how best health‐care users can participate and be actively engaged, it is important to consider the intended outcome, that is, the improved health and well‐being of the patient and/or the public, and how to collaborate to achieve this outcome.

One of the challenges in achieving patient and public involvement (PPI) in development of services is recognizing the opportunities. These opportunities and therefore how best to involve patients and the public will depend to some degree on the context or the stage of the person's journey. This journey often begins with assessments, as seen in a publication in this issue of HEX. In a systematic review of qualitative studies investigating adults' experiences of being assessed for psychological therapies, Sweeney et al analysed the findings according to the person's journey of being assessed, at the assessment stage and after the assessment. The themes and subthemes identified demonstrated the emotional impact that assessments can have (amongst other findings) at each stage of the journey. The insights highlight the importance of a therapeutic alliance and effective communication with people about their assessment and results as well as the therapies. Furthermore, the findings indicate the opportunities for active involvement of people in the assessment process.

One approach to identifying opportunities is to review existing approaches to PPI in health service development. Lalani et al conducted a systematic review of PPI in medical processes, namely processes involving doctors. A broad range of studies were identified across 10 countries, revealing the predominant methods of PPI as receiving feedback or complaints from patients. The authors identified the gaps in PPI in medical processes and opportunities in identifying and shaping the PPI agenda in this area.

Too often, interventions delivered by health‐care professionals do not produce the intended outcome. Whilst many factors may be responsible, limited patient or public involvement in intervention development may account for some of these observations. Tonge et al evaluated the acceptability of a targeted approach to early diagnosis of lung cancer in “ever‐smokers.” This qualitative study revealed two main themes centred around the acceptability of lung screening, and participants' desire to know about their own lung health via screening. Similar to other research, Tonge et al found that the participants' decision making regarding screening was influenced by a number of factors. The authors have presented a “push and pull” effect on the decision making, based on their findings, which clearly demonstrates the significance of patient involvement and shared decision making surrounding screening.

In a qualitative evaluation of the intersectoral strategies (between education and health) to address sexual and reproductive health, and pregnancy amongst adolescents in Chile, Obach et al reported that school‐based strategies which include access to contraception address adolescents' needs better than health centre‐based strategies which are limited in the quality time and frequency needed to establish and nurture relationships with this group.

In another qualitative study evaluating users' experiences of a Norwegian health centre delivering services to improve a healthy lifestyle and reduce the risk of non‐communicable diseases for individuals accessing the centre, Sagsveen et al have demonstrated the importance of evaluating the user's perspectives when determining the impact of the services delivered. Factors such as trust, ownership over goal setting and involvement in group activities influenced users' involvement in their own lifestyle change processes.

Richardson et al also evaluated the role of service users and the public, however, regarding health and social care regulation. From this qualitative study, the authors concluded that building ongoing relationships would create opportunities for continued service user and public involvement.

Bilodeau and Tremblay used an interprofessional patient‐centred (IPPC) practice framework to assess and describe the IPPC practice of oncology teams, with a view to determine whether the framework was appropriate and useful in describing IPCC practice. The authors provide an alternative perspective to IPPC practice to better understand the patient‐centred process in oncology teams.

To determine the success of involving and engaging patients and the public at every step of health care requires the use of valid and reliable measures. Availability and access to valid and reliable tools to measure the implementation of PPI or other concepts such as shared decision making and patient‐centred care remain a challenge for researchers and practitioners. In this issue of HEX, Ruiz Yanzi and colleagues report the validation of two questionnaires on shared decision making which they have translated and transculturally adapted to Spanish. Van der Weijden et al describe an 8‐step guidance for the development, content and governance of patient‐directed tools. A multi‐stakeholder collaborative process was utilized to develop and reach consensus regarding this guidance.

In our evolution of actively and effectively involving and engaging patients and the public at all stages of health care, we are likely to see more research and health‐care service delivery that includes the patient and/or the public in needs analysis, instrument development and validation, intervention and service development, quality assurance and feedback, and other key stages. HEX remains keen to publish manuscripts which will contribute to the growing evidence base on the PPI contribution to health service development and pathways for care.

